# Cognitive Processes Related to Memory Capacity Explain Nearly All of the Variance in Language Test Performance in School-Age Children With and Without Developmental Language Disorder

**DOI:** 10.3389/fpsyg.2021.724356

**Published:** 2021-09-21

**Authors:** Ronald B. Gillam, Sarfaraz Serang, James W. Montgomery, Julia L. Evans

**Affiliations:** ^1^Emma Eccles Jones Early Childhood Education and Research Center, Department of Communicative Disorders and Deaf Education, Utah State University, Logan, UT, United States; ^2^Department of Psychology, Utah State University, Logan, UT, United States; ^3^Department of Communication Sciences and Disorders, Ohio University, Athens, OH, United States; ^4^School of Behavioral and Brain Sciences, University of Texas-Dallas, Richardson, TX, United States

**Keywords:** developmental language disorder, cognition, memory, attention, language

## Abstract

The purpose of this study was to investigate the dimensionality of the cognitive processes related to memory capacity and language ability and to assess the magnitude of the relationships among these processes in children developing typically (TD) and children with developmental language disorder (DLD). Participants were 234 children between the ages of 7;0 and 11;11 (117 TD and 117 DLD) who were propensity matched on age, sex, mother education and family income. Latent variables created from cognitive processing tasks and standardized measures of comprehension and production of lexical and sentential aspects of language were tested with confirmatory factor analysis (CFA) and structural regression. A five-factor CFA model that included the constructs of Fluid Intelligence, Controlled Attention, Working Memory, Long-Term Memory for Language Knowledge and Language Ability yielded better fit statistics than two four-factor nested models. The four cognitive abilities accounted for more than 92% of the variance in the language measures. A structural regression model indicated that the relationship between working memory and language ability was significantly greater for the TD group than the DLD group. These results are consistent with a broad conceptualization of the nature of language impairment in older, school-age children as encompassing a dynamic system in which cognitive abilities account for nearly all of the variance in linguistic abilities.

## Introduction

There has been increased interest in the underlying nature of language impairments in children who present with deficits in the acquisition and use of morphosyntax, semantics, vocabulary, phonology/articulation and complex syntax (Tager-Flusberg and Cooper, 1999; Tomblin and Zhang, 1999; Bishop, 2014). Previously, researchers used the term specific language impairment (SLI) to refer to children with deficits that were specific to the “language” system (Adani et al., 2014). The term developmental language disorder (DLD) is now preferred because most children with language impairments display a variety of cognitive deficits in addition to their language difficulties (Bishop, 2014). For continuity and consistency, we use the term “DLD” when discussing previous research on children with language impairments regardless of whether the original paper referred to them as SLI or DLD. Many children with DLD have cognitive difficulties that include reduced phonological short-term memory (Gathercole and Baddeley, 1990; Gillam et al., 1998), slower speed of processing (Kail, 1994; Windsor and Hwang, 1999; Miller et al., 2001; Leonard et al., 2007), reduced verbal working memory (Ellis Weismer et al., 1999; Montgomery, 2000; Archibald and Gathercole, 2007), and poor controlled attention (CATT), including sustained attention (Sust Attn) (Spaulding et al., 2008; Finneran et al., 2009).

### Studies of Cognition and Language in Children With Developmental Language Disorder

The overwhelming majority of the studies documenting the cognitive and linguistic problems in children with DLD have employed quasi-experimental designs intended to assess potential differences in the distributions around group means on a few specific aspects of cognition and/or memory. Despite our knowledge about the range of cognitive processing limitations experienced by children with DLD, we know very little about the covariance structure or interrelatedness of their various cognitive and linguistic abilities or which cognitive processes are integral to language. This is an important question because it has implications for understanding the nature of language disorders, and it can inform language assessment and intervention procedures.

In the only large-scale investigations of the dimensionality and/or independence of cognitive processing abilities in school-age children that we are aware of, Leonard et al. (2007) administered a variety of memory and processing speed tasks to 204, 14-year-old children: 78 with language impairments and 126 who were typically developing. Participants completed five non-linguistic speed of processing tasks, four linguistic speed of processing tasks, four verbal working memory (WM) tasks, and one non-verbal WM task. The authors used confirmatory factor analysis (CFA) to assess the dimensionality of these different tasks followed by latent variable regression to determine the magnitude of the functional relationships between cognition and language. The best fitting CFA model, which was conducted on the TD and DLD groups combined, included 6 factors: general speed, non-linguistic speed, linguistic speed, verbal WM, non-verbal WM, and composite language. Regression analyses revealed that these cognitive factors accounted for 62% of the variance in language test scores. However, the only significant predictors were general speed (Standardized Beta = 0.17) and verbal WM (Standardized Beta = 0.73). The Leonard et al. (2007) study demonstrated that cognitive processing abilities related to verbal WM contributed to overall language scores on an omnibus measures of language comprehension and production to a much greater extent than cognitive processing abilities related to speed of processing and non-verbal WM.

In a smaller-scale study that addressed similar issues, Conti-Ramsden et al. (2015) gave working memory, procedural LTM, declarative LTM, and receptive grammar tasks to 45 children with DLD and 46 TD children between the ages of 8 and 14 years (*M* = 9;10). Working memory was assessed with the listening recall (remembering the last word in sentences after judging their accuracy), counting recall (counting dots in various arrays and then recalling the total number of dots in each array), and backward digits recall (repeating digits in reverse order) subtests from the *Working Memory Test Battery for Children* (*WMTB-C*; Pickering and Gathercole, 2001). All three complex WM tasks required children to retain auditory information in memory while performing some additional mental processing. Conti-Ramsden et al. found that their WM procedural memory and declarative memory measures accounted for 51.6 percent of the variance in the Test for Reception of Grammar 2nd Edition (TROG-2, Bishop, 2009). There were important group differences, with procedural memory predicting receptive grammar only for the TD group, while WM and declarative memory were predictive of receptive grammar only in the DLD group. In comparison to the Leonard et al. investigation, this study had many fewer participants, included fewer cognition and memory tasks, and only measured one aspect of language (grammatical comprehension) as the outcome. However, one advantage of this study over the Leonard et al. investigation was that these authors included procedural and declarative LTM tasks.

The current extended replication study was conducted to further explore two important issues that were raised by Leonard et al. (2007). The first issue relates to the importance of a variety of cognitive processes related to memory capacity, defined broadly. In the Leonard et al. (2007) study, four measures comprised the verbal WM latent factor [auditory WM, non-word repetition (NWR), competing language processing, and grammatical judgment listening span]. These four verbal WM measures accounted for more of the variance in children’s overall score on a language test than all the other cognitive and speed of processing measures combined. However, the four memory measures did not represent generally accepted components of verbal WM. In the current study, we selected cognitive processing and memory tasks that represented critical components of memory capacity, including LTM, as we explain further in the next section.

The second issue we explored in greater detail than Leonard et al. (2007) relates to potential differences in the interrelationships among cognition and language between children with and without DLD. Leonard et al. (2007) conducted their CFA and regression models on all the participants in their study (those with and without DLD) combined. They did that because they wanted to assess the dimensional nature of cognitive processes that relate to language performance across the full range of language ability. In our study, we used a combined-group approach as well as a multi-group approach that accounted for independent contributions of group variance in testing the model fit and in assessing potential group differences the magnitude of the relationships between cognition and language. We first specified a conceptual model of cognitive processes that mapped hypothesized memory constructs onto latent variables (sets of observed measures) that had the potential to be theoretically relevant to lexical and grammatical comprehension and production. In the second step, we translated our conceptual model into a measurement model to test the ability to derive a unique estimate of each construct. We used confirmatory factor analysis (CFA) to compare our original measurement model to two other nested models. Our primary research question was: Do measures of fluid intelligence, CATT, complex working memory, language knowledge in long-term memory, and language ability represent separate dimensions in a large sample of school-age children?

Next, we imposed successive restrictions on the best fitting CFA model to determine whether there were equivalent patterns of fixed and free factor loadings across the two groups. The research question was: Are the dimensions of cognitive processes and language ability invariant across children with and without DLD who were propensity matched on age, gender, parent education and family income? Finally, we used direct and indirect regressions to determine whether the magnitude of the relationships between memory processes and language ability differed in children with and without DLD. The specific research question was: Does the magnitude of the relationships between cognitive measures and standardized measures of language ability differ for children developing typically and children with DLD?

### Specification of the Conceptual Model

Our conceptual model represented a set of interactive components that have appeared in multiple models of memory capacity, and that have been shown to relate to language development and performance. The theoretically motivated components in our conceptual model of memory-related cognitive processes were fluid reasoning, CATT, WM, and language knowledge in LTM. Fluid reasoning refers to novel, non-verbal problem solving that involves concurrent information processing and storage. The storage demands on fluid reasoning tasks appear to reflect the ability to retain stimulus items that have been associatively “bound” together (Wilhelm et al., 2013). The relation between fluid intelligence and memory capacity may reflect the toggling of attention between the stimulus items and the processes of associatively binding items together (Shipstead et al., 2014, 2016). Evidence shows a moderate to strong relationship between fluid reasoning and WM in adults (Fukuda et al., 2010; Burgess et al., 2011) and children (de Abreu et al., 2010).

The second and third components in our conceptual model, CATT, and WM, are linked theoretically and empirically (Barrouillet and Camos, 2001; Baddeley, 2012; Baddeley et al., 2021; Cowan et al., 2021). CATT involves switching attentional focus between performing the processing activities of a cognitive task and maintaining items in storage. Working memory is the ability to hold information in an active state while some cognitive activity occurs (Baddeley et al., 2021; Cowan et al., 2021). Following research by Engle et al. (1999) and Mashburn et al. (2021) our WM latent variable was comprised of both simple (immediate) and complex recall tasks. We propose that many language activities involve the dual management of verbal processing and storage and thus, both attention control and WM processes (Marton and Schwartz, 2003; Lewis and Vasishth, 2005; Robertson and Joanisse, 2010). Tasks that involve simultaneous verbal-visual processing and/or storage may well require the use of CATT to manage these concurrent cognitive demands. For example, sentence comprehension and language production involve the control of concurrent verbal processing and storage in TD children (Adams and Gathercole, 1995) and children with DLD (Marini et al., 2014; Montgomery et al., 2018).

Finally, we included WM-LTM as a latent variable in our conceptual model because numerous models of memory capacity stress the importance of LTM (Baddeley et al., 2021; Cowan et al., 2021). Lexical knowledge in LTM has been shown to be a significant predictor of children’s verbal memory capacity (Montgomery et al., 2017). With respect to the relationship between the WM-LTM link and language performance, we propose that during language comprehension or production, individuals activate different units of knowledge or representations such as words or multi-word units (phrases, clauses) based on what they are hearing or want to say (Federmeier and Kutas, 1999; Acheson and MacDonald, 2009; Slevc, 2011). Because language comprehension and expression usually involve sentences and strings of connected sentences, individuals must be able to package these strings into fewer, but larger and more coherent/integrated strings or chunks. It is individuals’ language knowledge in LTM that allows them to perform this chunking function. The obvious cognitive payoff to knowledge-based chunking is the conservation of memory space in the moment. Results of recent sentence comprehension studies provide evidence of just such a relationship among WM, LTM, and language (Montgomery et al., 2018).

Previous research on cognition and language has mainly focused on the independent roles of CATT, phonological short-term memory (pSTM) storage, complex working memory (cWM) involving concurrent storage and processing, and processing speed. In the present study, we expand these cognitive mechanisms to also include LTM, and we study them together in a dynamic system. Accordingly, our first aim was to construct a conceptual model of the cognitive factors underlying memory capacity in school-age children. The second aim was to investigate the dimensionality of cognitive processes and language ability and to determine whether the dimensions are invariant across children with and without DLD. The third aim was to assess potential group differences in the magnitude of the relationships between measures of cognitive processing and language ability.

## Materials and Methods

### Participants

Participants in this study were 234 children between the ages of seven and 11 years: 117 with DLD (*Age*_*M*_ = 9:5) and 117 TD children (*Age*_*M*_ = 9:5). Children were recruited from various school systems, community centers, and university-sponsored summer camps for children four regions of the United States: Athens, Ohio; Logan, Utah; San Diego, California; and Dallas, Texas. The study was approved by the Internal Review Boards at four research universities, and the parents of all participants signed consent forms allowing their participation. All participants had: (a) normal-range hearing sensitivity bilaterally for the frequencies 500 Hz through 4 kHz (American National Standards Institute, 1997); (b) normal-range articulation on the word articulation subtest of the *Test of Language Development-4* (Newcomer and Hammill, 2008); and (c) normal or corrected vision. No participant had a history of neurological impairment or psychological/emotional disturbance, based on parent report. The degree of exposure to a second language was strictly controlled, with English being the primary language spoken by all the children. We excluded any child who spoke more than an average of 30 min of another language in the home or at school each day.

Similar to Leonard et al. (2007) study, lexical aspects of language ability were measured with the receptive and expressive portions of the *Comprehensive Receptive and Expressive Vocabulary Test* (CREVT-2; Wallace and Hammill, 1994), which has been demonstrated to be sensitive to breadth and depth of vocabulary knowledge in school-age children with and without DLD (McGregor et al., 2013). Grammatical aspects of language ability were measured with the Concepts and Following Directions subtest and the Recalling Sentences subtest of the *Clinical Evaluation of Language Fundamentals* (CELF-4; Semel et al., 2003).

Children were classified as DLD if the mean composite language z-score on the three lowest measures of lexical and grammatical aspects of language ability was at or below −1SD. Children were defined as TD if the mean composite language z-score on the three lowest measures of lexical and grammatical aspects of language ability was greater then −1SD. This approach, which diminished the influence of outlier scores in a single language domain, yielded a sample of children who represented the full spectrum of impairment from mild to severe. Performance distributions of children with DLD were consistent with the DSM-5 definition of language disorder (Gillam et al., 2017) and with other multi-dimensional systems that have been used to define SLI (e.g., Tager-Flusberg and Cooper, 1999; Leonard, 2014).

Language data for the two groups and Cohen’s *d* effect sizes are presented in Table 1. The TD group attained a significantly higher score on each language measure (all with very large effect sizes): CREVT-R [*t*(216) = 9.7, *p* < 0.001]; CREVT-E [*t*(232) = 10.79, *p* < 0.001]; CELF concepts and following directions [*t*(193) = 9.8, *p* < 0.001]; and CELF recalling sentences [*t*(232) = 15.04, *p* < 0.001]. In our study, the average composite z-score of the language measures was −1.48 (SD = 0.39; range = −2.73 to −1.00) for the DLD group and 0.08 (SD = 0.60; range = −0.96 to 1.89) for the TD group. These group distributions and differences are nearly identical to those of the participants in the Leonard et al. (2007) study. Their participants were a subset of the children from a large epidemiological study by Tomblin et al. (1997) in which children were diagnosed as SLI when their scores on two or more composites (vocabulary, sentence, narration, language comprehension, language production) were 1.25 or more standard deviations below the mean. In fact, Tomblin et al. (1997) reported that the average language z-score for the children identified with the EpiSLI model was −1.14, and approximately five percent of their SLI group had average z-scores between −1 and 0. In our study, the average composite z-score for the children in our DLD group was −1.48 with a SD of 0.39 (range = −2.73 to −1.00). None of the children in our DLD group had an average z-score between −1 and 0. Additionally, like the children in the Leonard et al. (2007) study, the overwhelming majority of the children in our DLD group (84.6%) had mixed receptive-expressive disorders. A few children (14.5%) exhibited expressive-only disorders, and just 1% exhibited receptive-only disorders.

**TABLE 1:** Performance on the language and cognitive measures for the children with developmental language disorder (DLD) and typically developing (TD) controls, and Cohen’s *d* (standardized mean difference) between the groups.

	**DLD**	**TD**	
**Factors/Measures**	**(*N* = 117)**	**(*N* = 117)**	**Cohen’s *d***
**Lexical Language**			
*CREVT-R Receptive^1^*			
*M*	30.89	44.87	1.27
*SD*	9.43	12.39	
*CREVT-R Expressive^2^*			
*M*	8.83	15.03	1.44
*SD*	4.03	4.72	
**Sentential Language**			
*CELF-4 Concepts and Follow Directions^3^*			
*M*	38.57	47.87	1.28
*SD*	8.77	5.43	
*CELF-4 Recalling Sent^4^*			
*M*	41.43	65.31	1.97
*SD*	11.24	12.97	
**Fluid Reasoning**			
*Leiter Figure Ground^5^*			
*M*	21.10	23.11	0.53
*SD*	3.53	4.07	
*Leiter Sequential Order^6^*			
*M*	28.47	34.14	0.64
*SD*	9.78	7.92	
*Leiter Repeated Patterns^7^*			
*M*	19.68	21.95	0.56
*SD*	3.84	4.26	
**Controlled Attention**			
*Sustained Attention^8^*			
*M* (P_*r*_ × 100)	76.21	81.45	0.31
*SD*	19.18	14.11	
*Attention Switching^9^*			
*M (percent correct)*	79.85	86.80	0.51
*SD*	14.79	11.86	
**Working Memory**			
*Digit Recall^10^*			
*M (trials correct)*	8.70	11.08	0.93
*SD*	2.02	3.02	
*Non-word Repetition^11^*			
*M* (*percen*t phonemes correct)	80.89	90.78	0.95
*SD*	13.42	6.16	
*Hi-Low^12^*			
*M (percent trials correct)*	54.55	72.05	0.90
*SD*	19.32	19.57	
*WJ-AWM^13^*			
*M* (memory span)	2.58	3.79	0.99
*SD*	1.19	1.26	
**Long-Term Memory – Language Knowledge**			
*TNL McDonald’s Comprehension^14^*			
*M (raw score)*	9.54	11.86	1.01
*SD*	2.37	2.21	
*TNL McDonald’s Retell^15^*			
*M (raw score)*	9.97	16.36	1.28
*SD*	4.85	5.14	

*^1^Comprehensive Expressive-Receptive Vocabulary Test-Revised: receptive subtest raw score.*

*^2^Comprehensive Expressive-Receptive Vocabulary Test-Revised: expressive subtest raw score.*

*^3^Clinical Evaluation of Language Fundamentals-4th Ed: concepts and directions raw score.*

*^4^Clinical Evaluation of Language Fundamentals-4th Ed: recalling sentences raw score.*

*^5^Leiter International Performance Scale-R: figure-ground subtest raw score.*

*^6^Leiter International Performance Scale-R: sequential order subtest raw score.*

*^7^Leiter International Performance Scale-R: repeated patterns subtest raw score.*

*^8^Sustained Attention: P_*r*_ discrimination index (P_*r*_ = Hits – False Alarms).*

*^9^Auditory Attention Switching: percent trials correct.*

*^10^Digit Recall: total number of lists recalled correctly.*

*^11^Non-word Repetition Task: percent phonemes correct.*

*^12^Hi-Low Task: percent trials correct.*

*^13^Auditory Working Memory subtest of the Woodcock-Johnson Cognitive Battery (3rd Ed): total raw score.*

*^14^Test of Narrative Language: total raw score on the McDonald’s Comprehension Subtest.*

*^15^Test of Narrative Language-Expressive: total raw score on the McDonald’s Retell subtest.*

### Propensity Matching

The DLD and TD groups were propensity matched on factors known to influence the language abilities of children. Propensity matching is a quasi-experimental approach used to equate groups on multiple categorical and continuous variables. It has been employed in clinical research to approximate the conditions of a randomized experiment by creating control and experimental groups that are balanced on a variety of variables (Rosenbaum and Rubin, 1983; D’Agostino, 1998). We calculated a propensity score for each child from a larger pool of 383 children (127 DLD, 256 TD) who completed the testing. Each child’s propensity score represented the conditional probability of that child being enrolled in the DLD or control (TD) group given the key baseline characteristics of age, sex, mother’s education level, and family income. Mother’s education and family income were used as proxy measures of socio-economic status (Shavers, 2007). The nearest neighbor matching method was used to match individual children with DLD to a typically developing counterpart who was observationally equivalent (i.e., had a similar propensity scores within a small range). Our propensity matching yielded samples of 117 children in each group. The DLD group was 57% male compared to the TD group, which was 63% male. The DLD group was 61% white (non-Hispanic), 12% Hispanic, 10% African-American, 10% more than one race, 4% Asian, and 3% American Indian/Native Hawaiian. The TD group was 72% white (non-Hispanic), 12% Hispanic, 0% African-American, 9% more than one race, 4% Asian, and 3% American Indian/Native Hawaiian. Non-parametric analyses revealed the groups were not significantly different with respect to age, gender, ethnicity, mother’s education, or family income.

### General Testing Procedure

The children were seen individually for administration of the standardized testing and experimental tasks. The order of the standardized assessments and experimental tasks were counterbalanced across visits and participants. The auditory tasks were presented under noise-reduction headphones at a listening level of dB SPL 55–75. All the children successfully completed practice trials prior to moving to the experimental portion of each task. E-Prime. v1 (Schneider et al., 2002) was used to deliver the tasks and to collect the children’s responses. Digital recordings were made for those tasks requiring a verbal response. Ten percent of the participants (equal numbers of children with DLD and TD children) from each of the three testing sites were selected at random to establish scoring reliability between two independent listeners.

### Latent Constructs and Measures Comprising the Cognitive Model

#### Fluid Reasoning

Three non-verbal measures were used to form the latent variable representing fluid reasoning. The primary executive abilities of interest were the ability to recognize patterns, to reason, and to solve novel problems independent of prior knowledge.

##### Figure-Ground (Leiter-FG)

The figure-ground subtest of the *Leiter-R* (Roid and Miller, 1997) served as an index of children’s ability to recognize and use holistic patterns in the service of solving a visual problem that involved identifying figures or designs that were embedded within complex stimuli. The dependent variable was the total number of items correct. Internal consistency reliability, as reported in the manual, varied from 0.74 to 0.80 across the ages of the children in this study.

##### Sequential Order Processing (Leiter-SO)

The sequential order subtest of the *Leiter-R* (Roid and Miller, 1997) served as an index of children’s ability to recognize and use serial order patterns in the service of solving a visual processing task. The assumption was that serial order processing was required because children look at logical progressions of pictures or figures and select an item that goes next in the progression. The dependent variable was total number of items correct. Internal consistency reliability, as reported in the manual, varied from 0.71 to 0.81 across the ages of the children in this study.

##### Repeated Patterns (Leiter-RP)

The repeated patterns subtest of the *Leiter-R* (Roid and Miller, 1997) represented the third measure of focal attention. Children supplied the missing portion of a pattern of pictures or figures by moving cards into an easel. Children needed to attend to the pictures and mentally represent the pattern, then shift their attention back and forth from the examiner’s model to the answer materials to select a card that fit the pattern. The dependent variable was total number of items correct. The internal consistency reliability across the ages of the children in this study varied from 0.70 to 0.81.

#### Controlled Attention

Two auditory measures were used to form the latent variable representing CATT. The primary executive abilities of interest were the ability to focus attention, to sustain attention, to divide attention, and to switch attention during pattern recognition tasks.

##### Sustained Attention

Children completed a sustained auditory attention measure adapted from the auditory vigilance subtest of the *Gordon Diagnostics System* (Gordon et al., 1997). Children were instructed to hold a very simple pattern in mind (the number sequence 1–9) and respond each time they heard pattern within a running stream of random numbers. The task required children to maintain attention over the course of 10 min and respond only when they heard the two-digit sequence (1–9). Internal consistency reliability was 0.88. Scoring reliability agreement was 100% between the initial coding and re-analyzed coding of all trials.

##### Attention Switching

An auditory switching task (Evans et al., 2018) was used to index children’s auditory attention switching (AttSW) abilities. The task was modeled after a two-speaker dichotic listening task designed by Ross et al. (2010). The stimuli consisted of male and female speakers saying numbers and letters. The auditory stimuli were letters (A-E) and numbers (1–5) generated from digitally created recordings using the AT&T speech generator. A man’s voice was played in one ear and a woman’s voice in the other ear and each voice was saying numbers or letters at the same time. The number and letter items were paired such that children only heard a number in one ear and a letter in the other ear, never a number or letter in both ears. A beep sounded periodically in one ear or the other signaling which side to pay attention to. Immediately after hearing the tone, children were instructed to touch the blocks of letters or numbers on the screen representing the patterns they were listening to (letters or numbers) in the ear that the tone was in. The presentation of male/female speakers to the left/right ears was counterbalanced across children to control for any possible speaker or preferred ear bias. Trials were presented in a fixed random order. The primary dependent variable was total accuracy. Internal consistency reliability was 0.94. Coding agreement between the initial coding and re-analyzed coding of correct switch trials was 100%.

#### Working Memory

Two simple (immediate) recall tasks and two complex recall tasks that combined immediate and concurrent memory storage and cognitive processing were used to form the WM latent variable.

##### Non-word Repetition

Children completed the NWR task developed by Dollaghan and Campbell (1998). Each phoneme within each word was scored as correct or incorrect relative to the target phoneme. Phoneme additions, substitutions and omissions were scored as errors. The number of phonemes repeated correctly was then divided by the total number of phoneme targets, yielding a percentage of phonemes correct (PPC) for each syllable length. The dependent variable was PPC. Internal consistency reliability was 0.83. Item transcription and scoring reliability were at or above 0.95.

##### Digit Recall (DigitMem)

Children completed a conventional digit recall task in which they were told that they would hear a man saying lists of numbers and were asked to repeat as many of the digits in each list in the same order they heard them. The dependent variable was total number of trials correct. Internal consistency reliability was 0.87. Item transcription and scoring reliability were at or above 0.97.

##### Woodcock-Johnson III Auditory Working Memory

Children completed the standardized auditory working memory subtest of the *Woodcock-Johnson III* (Woodcock et al., 2001). Stimulus items included the digits one through nine and 50 words. Children listened to a male voice saying a list of words and numbers (*4, orange, 1, bear, 7*). After each list they repeated the words in serial order (*orange, bear*) followed by the digits in serial order (*4, 1, 7*). The storage component involved the children remembering the items and the processing component entailed their organizing the items during recall into words and digits. The primary dependent variable was total number of trials correct. Internal consistency reliability, as reported in the manual was 0.86. Item transcription and scoring reliability were at or above 0.97.

##### High-low Task

Children completed an experimental WM task developed for children to assess the coordination of storage and processing (Magimairaj and Montgomery, 2012, 2013). Prior to each tone, a fixation point appeared on the screen for 150, 300, or 600 ms (random across trials). After reporting the new count of high tones and low tones, children pressed the space bar for the next tone. At the end of each block, the monitor turned green cueing children to verbally report each count. Each trial sequence consisted of seven to 11 tones with six trials at each sequence length, for a total of 30 trials. The storage component was retaining the number of high and low tones heard and the processing component related to the children determining whether a tone was high or low. The primary dependent variable was percent trials count. The internal consistency reliability was 0.84. There was 100% agreement between the initial coding and re-analyzed coding for count scores.

#### Long-Term Memory – Language Knowledge

Story retelling tasks have long been used to represent episodic memory (Christoffels, 2006; Bower, 2008; Kapikian and Briscoe, 2012). We administered the McDonalds comprehension and story retell tasks from the Test of Narrative Language (TNL: Gillam and Pearson, 2004) to represent the episodic, lexical-semantic and grammatical aspects of extant language knowledge within LTM. These tasks require the child to remember specific details of a story that is told to them and then to use their long-term knowledge of narrative structure and sentence structure to retell the story. Performance on these tasks is dependent upon bindings from the activation of lexical items and relevant syntactic processing schemes.

##### Story Recall (TNL-Rec)

Children listened to the McDonald’s recorded story from the *TNL*. Immediately following the story, story recall was assessed by a set of literal and interferential questions. The dependent variable was total raw score. Internal consistency reliability, as reported in the test manual, was 0.87. Scoring reliability between two independent listeners was 0.97.

##### Story Retelling (TNL-Retell)

A narrative retell task followed the recall task. The story was scored for key words from the stimulus story that were retained in the retelling. The dependent variable was the total raw score. Internal consistency reliability, as reported in the test manual, was 0.89. Transcription and scoring reliability between two independent listeners was 0.91.

### Data Analysis

**FIGURE 1:**
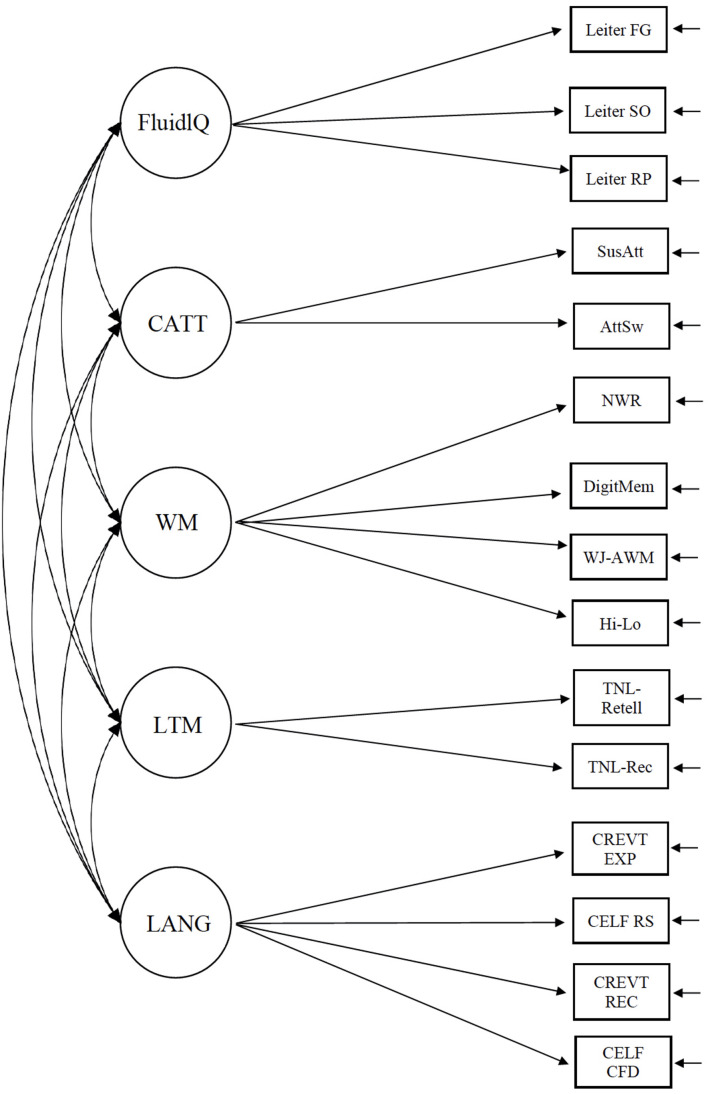
The five-factor conceptual model containing the latent variables of fluid reasoning (FLDR), controlled attention (CATT), working memory (WM), long-term memory – language knowledge (LTM-LK) and language (LANG).

**FIGURE 2:**
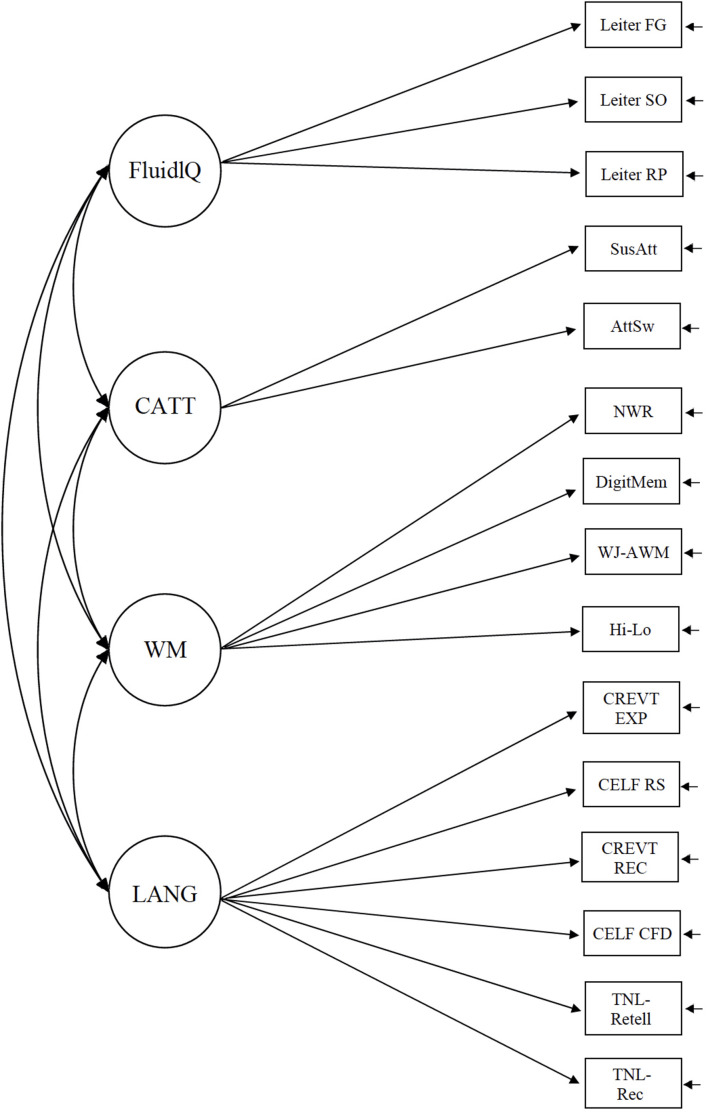
A four-factor conceptual model in which the LTM-LK measures were embedded in the language latent variable.

**FIGURE 3:**
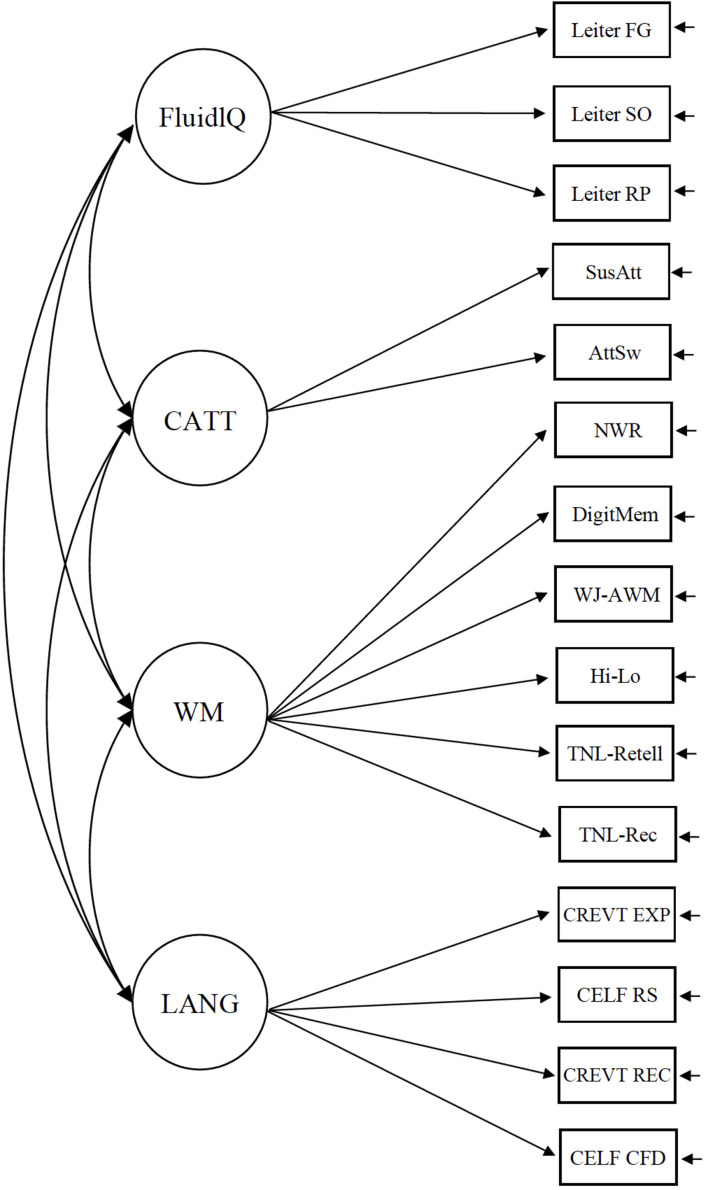
A four-factor conceptual model in which the LTM-LK measures were embedded in the working memory latent variable.

All models were fit in M*plus* (Muthén and Muthén, 2017). We first specified a conceptual model of cognitive processes that mapped hypothesized constructs related to memory onto latent variables (sets of observed measures) that had the potential to be theoretically relevant to language abilities (Model 1, Figure 1). Our five-factor model consisted of four cognitive factors (Fluid Reasoning, CATT, Working Memory, and LTM-LK) and a language factor. In the second step, we examined whether our measurement model was the most appropriate conceptualization for our data. As noted in the introduction, language plays a role in most measures of WM and LTM. We wondered whether our measures of LTM-LK were better characterized as part of the language factor. We compared the model fit of our five-factor CFA model (Model 1) to a nested four-factor model in which the two LTM-LK measures (TNL-recall and TNL-retelling) were included as part of the Language latent variable (Model 2, Figure 2). One other possibility was that the two measures that comprised the LTM-LK factor were better characterized as part of the WM factor. We compared the model fit of our original five-factor CFA model (Model 1) to a nested four-factor model in which the two LTM-LK measures were included as part of the WM latent variable (Model 3, Figure 3). We fit each of these models to the full sample, combining TD and DLD groups, and used a Chi-square test to compare them. In the third step, we reran our best-fitting model as a multiple group model (Jöreskog, 1969), separating TD and DLD groups to ensure the model was appropriate when the groups were treated as distinct. We applied grand-mean centering to the observed scores and used maximum likelihood estimation for the models (maximum likelihood robust, or MLR, results were virtually identical). We did not test measurement invariance because we were not interested in comparing mean differences between latent variables across groups. If we had tested for measurement invariance, we expect that we would not find it, given that the groups were specifically constructed to differentiate between language ability.

After determining which measurement model was most appropriate for our data, we examined moderation with a multiple group framework for testing whether the regression coefficients relating the four cognitive factors (Fluid Reasoning, Attention, WM, and LTM-LK) to language ability measures differed across groups. We did this by fitting two nested multiple group models. In the first model, all parameters were constrained to be equal across groups. In the second model, all parameters except the four regression coefficients and the associated residual variance were constrained to be equal within the two groups. Thus, any differences between these two models could be attributed to unique differences between the two groups in the relationships between the four cognitive factors and language. The likelihood ratio test was used to compare these two models. A significant result would indicate that the relationships between the four factors and language differed between the TD and DLD groups.

We judged the goodness-of-fit of our models according to a combination of indices including the model chi-square test, Browne and Cudeck (1993) root mean square error of approximation (RMSEA), Bentler (1990) comparative fit index (CFI), and the Standardized Root Mean Square Residual (SRMR). The model Chi-square test assesses the magnitude of discrepancy (e.g., the badness of fit) between the sample and fitted covariance matrices (Hu and Bentler, 1999). An insignificant result indicates a good model fit (Barratt, 2007). However, the chi-square statistic is sensitive to sample size, so it often rejects models when there are large sample sizes (Bentler and Bonnet, 1980), as is the case of our investigation. When comparing models derived from large samples, the best model is usually considered to be the one with the smallest chi-square value. The RMSEA (Steiger, 1990) assesses the extent to which the model fits the population’s covariance matrix. An RMSEA of less than 0.07 indicates an acceptable fit, and an RMSEA of 0.05 or less indicates a good fit (Browne and Cudeck, 1993). The CFI compares the chi-square value of the model to that of a null model (the worst-case scenario). CFI values at or above 0.95 indicate a good model fit (Hu and Bentler, 1999), with values above 0.97 indicating a very good fit. The SRMR indicates how well sample variances, covariances, and means were reproduced by the model. A CFI greater than 0.95 in combination with an SRMR less than 0.08 are reasonable cut-off values for good fitting models (Hu and Bentler, 1999).

## Results

Descriptive statistics for each group and group comparisons are provided in Table 1. Preliminary *t*-tests revealed that the TD group earned significantly higher scores than the DLD group on all the language and cognitive processing measures at the *p* < 0.001 level with the exception of one measure, Sust Attn, which yielded a *p* < 0.05. The small probability values are a function of the large sample size and are minimally informative. We therefore calculated Cohen’s *d* standardized mean difference scores to better represent the extent of group differences on the cognitive measures in the three models. The group differences for all the language tasks, the WM tasks and the LTM-LK tasks were large (Language *d* = 1.27–1.97, WM *d* = *0.90* –0.99, LTM-LK *d* = 1.01–1.28); those for our Fluid Reasoning tasks were all in the moderately large range (*d* = 0.53 −0.64); and those for the CATT tasks were in the moderate-to-moderately large range (*d* = 0.31 −0.51).

Correlation matrices for the observed measures for the two groups combined are provided in Table 2. All the Pearson bivariate correlations were statistically significant, *p* ≤ 0.001. The highest correlations tended to occur among the language subtests (0.60–0.75) and the cognitive measures comprising each of the six latent variables (*r* = 0.40 −0.59). Measures across the latent variables were generally correlated in the moderate to moderately high range (*r* = 0.33 to 0.55). Looking at the correlations among the five latent variables themselves (Table 3), note that fluid reasoning was highly correlated with WM (*r* = 0.82), while language was highly correlated with fluid reasoning (*r* = 0.82), WM (*r* = 0.91), and LTM-LK (*r* = 0.85).

**TABLE 2:** Pearson correlations among the cognitive processing measures for the two groups combined.

	**1**	**2**	**3**	**4**	**5**	**6**	**7**	**8**	**9**	**10**	**11**	**12**	**13**	**14**	**15**
1 Leiter-FG	1	0.519**	0.406**	0.364**	0.213**	0.425**	0.435**	0.258**	0.289**	0.344**	0.269**	0.451**	0.498**	0.462**	0.444**
2 Leiter-SO	0.519**	1	0.428**	0.407**	0.334**	0.444**	0.524**	0.319**	0.349**	0.377**	0.338**	0.474**	0.516**	0.499**	0.487**
3 Leiter-RP	0.406**	0.428**	1	0.318**	0.267**	0.411**	0.461**	0.282**	0.314**	0.349**	0.366**	0.396**	0.499**	0.409**	0.393**
4 Sust Attn	0.364**	0.407**	0.318**	1	0.444**	0.362**	0.456**	0.334**	0.316**	0.225**	0.233**	0.371**	0.352**	0.366**	0.409**
5 Attn Switch	0.213**	0.334**	0.267**	0.444**	1	0.306**	0.391**	0.301**	0.219**	0.223**	0.280**	0.361**	0.337**	0.353**	0.367**
6 WJAWM	0.425**	0.444**	0.411**	0.362**	0.306**	1	0.557**	0.411**	0.511**	0.481**	0.452**	0.509**	0.586**	0.608**	0.573**
7 High-Low	0.435**	0.524**	0.461**	0.456**	0.391**	0.557**	1	0.428**	0.521**	0.456**	0.440**	0.488**	0.579**	0.584**	0.539**
8 NWR	0.258**	0.319**	0.282**	0.334**	0.301**	0.411**	0.428**	1	0.438**	0.297**	0.290**	0.376**	0.385**	0.565**	0.388**
9 Digit Recall	0.289**	0.349**	0.314**	0.316**	0.219**	0.511**	0.521**	0.438**	1	0.392**	0.318**	0.440**	0.479**	0.657**	0.517**
10 Story Retell	0.344**	0.377**	0.349**	0.225**	0.223**	0.481**	0.456**	0.297**	0.392**	1	0.660**	0.600**	0.607**	0.656**	0.540**
11 Story Recall	0.269**	0.338**	0.366**	0.233**	0.280**	0.452**	0.440**	0.290**	0.318**	0.660**	1	0.502**	0.603**	0.576**	0.498**
12 CREVT EXP	0.451**	0.474**	0.396**	0.371**	0.361**	0.509**	0.488**	0.376**	0.440**	0.600**	0.502**	1	0.754**	0.689**	0.598**
13 CREVT REC	0.498**	0.516**	0.499**	0.352**	0.337**	0.586**	0.579**	0.385**	0.479**	0.607**	0.603**	0.754**	1	0.753**	0.640**
14 CELF RS	0.462**	0.499**	0.409**	0.366**	0.353**	0.608**	0.584**	0.565**	0.657**	0.656**	0.576**	0.689**	0.753**	1	0.700**
15 CELF CFD	0.444**	0.487**	0.393**	0.409**	0.367**	0.573**	0.539**	0.388**	0.517**	0.540**	0.498**	0.598**	0.640**	0.700**	1

*Leiter-FG, Leiter Figure Ground; Leiter-SO, Leiter Sequential Order; Leiter-RP, Leiter Repeated Patterns; Sust Attn, Sustained Attention; WJAWM, Woodcock Johnson Auditory Working memory; High-Low, High-Low task; NWR, Non-word Repetition; Digit Recall, Digit Recall task; Story Retell, TNL McDonald’s story retell; Story Recall, TNL McDonald’s story comprehension questions; CREVT EXP, CREVT Expressive Vocabulary Test; CREVT REC, CREVT Receptive Vocabulary Test; CELF RS, CELF-4 Recalling Sentences; CELF CFD, CELF-4 Concepts and Following Directions. ^∗∗^Correlation is significant at the 0.01 level (2-tailed).*

**TABLE 3:** Correlations among latent variables for the two groups combined (all correlations significant at *p* < 0.001).

	**Fluid Reasoning**	**Controlled Attention**	**Working Memory**	**Long-term Memory**	**Language**
Fluid Reasoning	1.00				
Controlled Attention	0.71	1.00			
Working Memory	0.82	0.72	1.00		
Long-term Memory	0.61	0.42	0.71	1.00	
Language	0.81	0.62	0.91	0.85	1.00

### Confirmatory Factor Analysis

Fitting our original five factor CFA model (Model 1) to the full data resulted in model fit of χ^2^(80) = 147.25, *p* < 0.001. Since χ^2^ test statistics tend to be too liberal in samples as large as ours, we also examined the AIC, RMSEA, the CFI, the TLI, and the SRMR. According to the cutoffs suggested by Hu and Bentler (1999), Model 1 had an acceptable fit (AIC = 21973.86; RMSEA = 0.06; CFI = 0.963; TLI = 0.951; SRMR = 0.036). Given the high correlations between the working memory, long-term memory, and language factors and the fact that all three constructs involve language components, we decided to test our original model against a model in which the two measures that originally comprised the LTM-LK factor were included in the Language latent variable (Model 2) and a model in which the two LTM-LK measures were included in the WM latent variable (Model 3). The fit statistics for Model 2 (AIC = 22030.21; χ^2^(84) = 184.59, *p* < 0.001; RMSEA = 0.072; CFI = 0.944; TLI = 0.931; SRMR = 0.041) were poorer than those for Model 1 as demonstrated by larger AIC and RMSEA values and a statistically significant χ^2^ difference test, χ^2^(4) = 37.39, *p* < 0.0001. Similarly, the fit statistics for Model 3 (AIC = 22034.47; χ^2^(84) = 215.85, *p* < 0.001; RMSEA = 0.082; CFI = 0.927; TLI = 0.909; SRMR = 0.046) were also poorer than those for Model 1 as demonstrated by larger AIC and RMSEA values and a statistically significant χ^2^ difference test, χ^2^(4) = 68.65, *p* < 0.0001. Finally, when we employed the multiple group approach to our best-fitting model (Model 1), the fit indices were slightly better than those for the two groups combined [AIC = 21795.80; χ^2^(180) = 254.26, *p* < 0.001; RMSEA = 0.059; CFI = 0.950; TLI = 0.942; SRMR = 0.063]. Notably, the fit of the five-factor model was significantly better when parameters in the model were allowed to vary independently for the two groups, χ^2^(55) = 432.61, *p* < 0.001.

### Structural Regression

**FIGURE 4:**
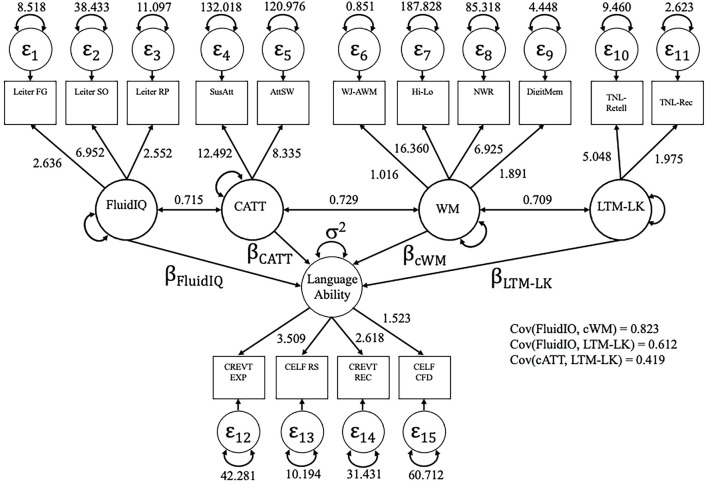
The five-factor measurement model containing the latent variables of fluid reasoning (FLDR), controlled attention (CATT), working memory (WM), long-term memory – language knowledge (LTM-LK) and language.

The structural regression model testing group differences in regression coefficients is given in Figure 4. The numerical parameter estimates provided are identical across groups because they were constrained to be so. The estimates not provided are the regression coefficients and the residual variance, which were allowed to differ across groups. These are given in Table 4. Based on the numerical values, these regression coefficients as a set appear to be different across groups, a conclusion verified by the likelihood ratio test, χ^2^(5) = 12.78, *p* = 0.026. The largest difference in parameters between groups was the effect of WM on language. Examining the standardized estimates, in the TD group, two people who are one standard deviation apart on WM are expected to be 0.839 standard deviations apart on language ability (*t* = 4.12, *p* < 0.001), whereas in the DLD group, two people who are one standard deviation apart on WM are expected to be only 0.067 standard deviations apart on language ability (*t* = 0.314, *p* = 0.753).

**TABLE 4:** Regression estimates and standard errors across groups from Figure 4.

		**TD**				**DLD**			
		**Estimate**	**Std. Error**	***t***	***p***	**Estimate**	**Std. Error**	***t***	***p***
Unstandardized	ß_*FluidIQ*_	0.123	0.867	0.142	0.887	1.014	0.568	1.785	0.074
	ß_*Att*_	–0.683	0.760	–0.900	0.368	0.748	0.517	1.448	0.148
	ß_*WM*_	4.114	1.052	3.911	<0.001	0.240	0.768	0.313	0.755
	ß_*LTM*_	1.523	0.119	2.437	0.015	2.006	0.426	4.714	<0.001
	σ^2^	0.941	1.098	0.857	0.391	0.892	0.649	1.374	0.169
Standardized	ß_*FluidIQ*_	0.025	0.177	0.141	0.888	0.284	0.158	1.798	0.072
	ß_*Att*_	–0.139	0.156	–0.895	0.371	0.210	0.148	1.419	0.156
	ß_*WM*_	0.839	0.204	4.120	<0.001	0.067	0.214	0.314	0.753
	ß_*LTM*_	0.274	0.109	2.510	0.012	0.562	0.109	5.170	<0.001
	σ^2^	0.039	0.045	0.871	0.384	0.070	0.048	1.448	0.148

*TD = ß_*FluidIQ*_, Beta value for Fluid Intelligence; ß_*Att*_, Beta value for Controlled Attention; ß_*WM*_, Beta value for working memory; ß_*LTM*_, Beta value for long term memory; σ^2^, residual variance for language ability.*

A formal hypothesis test of the effect of WM on language ability yielded a significant group difference at the 0.05 level, χ^2^(2) = 7.47, *p* = 0.024. This result could be conceptualized as a group by WM interaction, as the effects of WM on language ability differ by group. However, this finding should be considered tentative, since it is a *post hoc* test, and would not hold up in the presence of a multiple correction procedure such as a Bonferroni correction. Tests for group differences on effects of Fluid Reasoning χ^2^(2) = 2.71, *p* = 0.258, CATT χ^2^(2) = 1.35, *p* = 0.509, and LTM-LK χ^2^(2) = 1.51, *p* = 0.471 produced non-significant results. Finally, our four cognitive latent variables accounted for 96.1% of the variance in language ability in the TD group and 93.0% of the variance in the DLD group.

## Discussion

The overarching goals of this study were to investigate the dimensionality of cognitive processes and language ability, to determine whether the dimensions were invariant across children with and without DLD, and to assess potential group differences in the magnitude of the relationships between measures of cognitive processing and language ability.

The measures comprising our latent variables were designed to reflect important facets of the cognitive processes underlying memory capacity, and our formal language measures were well-designed assessments of lexical and sentential comprehension and production. The use of multiple measures for each construct increased the reliability of factor measurement compared with single-measure approaches (Kline, 2016). We took a psychometric approach with multiple steps that involved: (1) specifying a five-factor model of cognitive processing and language abilities; (2) translating our theoretical model into a statistical model; (3) using CFA to determine how well the model fit the cognitive data; and (4) using structural regression to test potential group differences. We believe this study meets the growing need for large-scale investigations that assess the nature of the relationships between cognition and language in older school-age children with DLD. Studies like this one benefit from validated, parsimonious measurement models that identify the types of measures that comprise the most salient characteristics of cognitive processing in children with and without DLD.

### Group Mean Differences on the Individual Measures

We found that the children in the TD group earned significantly higher scores than the children in the DLD group on all the language and cognitive measures that were administered, confirming the validity of the idea that DLD involves impairment of both cognition and language (Bishop et al., 2016). We calculated Cohen’s *d* standardized mean difference scores to represent the effect size of the group differences on the language measures. Group differences on the four lexical and sentential language measures were very large. With respect to the cognitive measures, we found large group differences (*d* values greater than −0.8) for the WM latent variable and the LTM-LK latent variable. There were moderate to moderately large group differences for the Fluid Reasoning latent variable and the CATT latent variable. These results are consistent with a broad conceptualization of the nature of language impairment, especially in older, school-age children, as encompassing a dynamic system that involves both cognitive and linguistic deficits.

### Working Memory and Language Knowledge in Long-Term Memory

In order to determine whether measures of cognition and language ability represented separate dimensions in a large sample of school-age children, we tested a conceptual model that was based on a broad perspective of the cognitive processes that have been studied with respect to cognitive capacity and language development. Our best-fitting model contained the latent variables of fluid intelligence, CATT, WM, language knowledge in LTM, and language. Fluid intelligence was a proxy for resource allocation among the abilities that are important for pattern recognition and non-verbal problem-solving. We reasoned that CATT (sustaining and switching attention) likely plays an important role in language development because it allows children to create and store in LTM-LK different kinds of linguistic representations (e.g., lexical, morphological, syntactic). For example, with respect to syntactic knowledge and sentence comprehension, AttSW may allow children to direct their attention between memory for storing intermediate structures and the language system to develop new structure from material downstream and to combine these structures into coherent syntactic-semantic representations. The importance of CATT processes to spoken word recognition and sentence comprehension has been demonstrated in studies of TD children (Finney et al., 2014) and in children with DLD (Montgomery, 2008; Evans et al., 2018; Montgomery et al., 2018).

Working memory, the third latent variable in the model, has been shown to play an important role in language development in children with and without DLD (Adams and Gathercole, 2000; Gathercole and Baddeley, 2014). Our WM latent variable was represented by two measures of phonological short-term memory (digit recall and NWR) that are commonly used to represent simple memory capacity and two measures of complex memory that had processing and storage components (WJ-AWM and a high tone – low tone counting and recall task). Phonological short-term memory has been shown to play a role in language, especially language comprehension, by non-typical adults (Wang et al., 2016) and by children with language impairments (Leonard et al., 2013). Complex working memory (cWM), representing the simultaneous cognitive processing and retention of information, plays an important role in language development because children must hold in mind representations words and sentences as they are trying to figure out what someone else is saying. With respect to language production, complex WM enables children to remember what they and others have said as they formulate and produce their own responses.

Language knowledge in long-term memory (LTM-LK) was the fourth latent variable in the model. We reasoned that one role of extant language knowledge in LTM is to organize and store integrated chunks of information for long periods of time. Language knowledge in LTM may be especially important for representing and activating intermediate products of processing (e.g., words and clauses) as well as the final product (a representation of one or more sentences). We used children’s performance on the two tasks related to the McDonald’s story on the *Test of Narrative* Language (Gillam and Pearson, 2004) to represent language knowledge in LTM. In these tasks, children listened to a story the examiner told about two children who went to McDonalds with their mother. Immediately after hearing the story, children completed a recall task in which they answered literal and inferential questions about the story. Children then completed a retell task that was scored according to the amount of information from the original story that was retained in their retelling. We decided these two tasks together would provide an index of language knowledge in LTM.

The fact that language and memory are both key components of remembering, understanding, and retelling stories was evidenced by high correlations between our WM, LTM-LK, and Language latent variables. We wanted to know whether our measures were better characterized as two factors (WM and Language) or three factors (WM, LTM-LK, and Language). When we compared the model fit statistics for our original five factor model to those for two nested, four-factor models (one with the LTM-LK measures included in the language factor and one with the LTM-LK measures included in the WM factor), it was clear that language knowledge in LTM was functionally separable from language and from WM. The two McDonald’s measures represented language knowledge in LTM because story recall and retelling are known to draw heavily on associative frameworks in episodic memory that are built up from prior experience listening to and telling stories (Bower, 2008; Anderson and Lebiere, 2014); the recall and retelling tasks required participants to retain information over a fairly long period of time (as opposed the immediate recall tasks we selected for assessing WM); and performance on both tasks benefited from the use of episodic encoding operations that integrate knowledge from semantic and syntactic language systems into multilevel representations.

Object knowledge, event knowledge, and language knowledge all reside in associative networks in LTM (Federmeier and Kutas, 1999; Lewis and Vasishth, 2005; Lewis et al., 2006; Woltz and Was, 2006; Chow et al., 2014). Cowan (1995, 2014) embedded processes model of WM posits that activation of information from an unlimited long-term memory (LTM) system in which cognitive strategies called *schemas* organize information. These schemas assist in regrouping or recoding multiple pieces of knowledge into coherent packages called *chunks* that become increasingly large and complex as new knowledge is added (Ericsson and Kintsch, 1995). Cowan’s notion of large, integrated chunks of information remaining in a state of activation in LTM for use by WM is consistent with our findings of language knowledge in LTM playing an important role in both WM and language.

### Dimensional and Categorical Characterizations of Developmental Language Disorder

The second goal of our study was to determine whether the cognitive and language dimensions that we verified were invariant across children with and without DLD. There is some debate as to whether DLD is better conceptualized as a dimensional disorder or a discrete, categorical disorder with its own phenotype and etiology (e.g., Dollaghan, 2004; Bishop, 2017). The dimensional view assumes that language ability falls along a wide continuum of linguistic and non-linguistic abilities, with the language abilities of children with DLD falling at the low end of the normal distribution (Leonard, 1991, 2014; Rescorla, 2009). A categorical view assumes that the observed symptoms of the disorder are qualitatively different from language delay with specific clinical markers. Our analyses allowed us to consider both a dimensional and categorical perspective by looking carefully at covariance among measures of cognition and language in children with and without DLD. Recall that the results of the confirmatory factor analysis (CFA) by Leonard et al. (2007) yielded very good model fit statistics for their DLD and TD groups combined. They combined their groups because the goal of their research was to take a dimensional view of childhood language disorders. Our results are in line with those of Leonard et al. (2007) in that our fit statistics also indicated a very good model fit when the DLD and TD groups were combined. Importantly, our findings extend those of Leonard et al. because our model was also a good characterization of the interrelationships among cognitive processes and language measures in the two groups separately. These results indicate that a model incorporating fluid intelligence, CATT, WM, and LTM-LK is a good characterization of cognitive abilities that are related to language measures in school-age children, whether they are DLD or TD. However, our structural regression analysis indicated that the specific effects of WM on language performance differed across the two groups, with WM predicting performance on our language measures better for the children in the TD group than the children in the DLD group. Our study does not indicate whether this difference is inherent in children with DLD or results from difficulties with both memory and language. Given group differences in the nature of the relationships between cognition and language, children with DLD may not merely be children who are otherwise normal except that their language scores fall at the low end of the normal language continuum. Instead, it may be better to conceive of children with DLD as a discrete clinical group with unusual relationships among cognition and language abilities that hinder development in a number of cognitive and linguistic domains simultaneously.

### Strengths of the Study

The present study investigated cognitive processes that were not just theoretically but also empirically relevant to children’s language performance. Doing so was important because it permitted us to determine whether the resultant model was not only relevant to the language performance of children overall but also to children with DLD separate from TD children. The fact that our model was invariant across the groups revealed the crucial importance of memory (WM, LK-LTM) to all children’s language performance. This finding advances our understanding of the relationship between cognition and language in school-age children. A second strength is that our groups were propensity matched on age, gender, and SES to control the potential effects of these variables. Propensity scores represent the probability of assignment to either the DLD or TD group based on a vector of observed covariates (Rosenbaum and Rubin, 1983; D’Agostino, 1998). This matching procedure allowed us to control for possible distortion of the results, both within and between groups, due to age, gender and SES. Propensity matching also allowed us to control for possible differences in participant enrollment strategies across the four research sites. In turn, we were able to address a counterfactual problem in many cross-sectional studies related to biased estimates of the relationships of interest when males and children from lower SES backgrounds are overrepresented in language impaired groups and underrepresented in typically developing groups. Our propensity matching procedure thus granted us a degree of confidence in the adequacy and applicability of our model for characterizing group differences in the relationships among cognition and language performance in school-age children, regardless of their sex and SES background.

### Limitations of the Study

We tested the dimensionality and group invariance of 13 cognitive processing and language measures using confirmatory factor analysis. There are three important caveats related to this investigation. First, while the five latent variables in our conceptual model were based on empirical and theoretical evidence, other conceptualizations of children’s cognitive and language abilities are possible. For example, we did not include measures of inhibition in our model. Second, there are measures other than the ones we selected that could represent our five factors. Different measures may have resulted in different model fit statistics, leading to a different final model. Third, we could have considered more restrictive criteria for differentiating children with and without DLD, which could have resulted in decreases in model fit. As noted by Kline (2016), regression analyses are more informative, and the results are more generalizable when the full range of ability within and across groups is represented.

### Summary

The current study took a psychometric approach to modeling a set of cognitive processing constructs that could conceivably influence children’s language performance. A five-factor model that included the latent variables of Fluid Intelligence, CATT, WM, Language Knowledge in LTM, and Language Ability fit the data significantly better than either of two nested, four-factor models in which LTM-LK was dropped and the two LTM-LK measures were integrated into either WM or language ability latent variables. In addition, the five-factor model fit significantly better when parameters were allowed to differ across the two groups. The group comparisons were based on a relatively large propensity-matched sample of children with and without DLD with equal probability of assignment to the DLD or TD groups based on age, sex, mother’s education and family income. Our overall model fit statistics suggest that measures of the lexical and grammatical language abilities and the cognitive processes related to memory capacity and language abilities constitute separate factors that covary to similar degrees in children with and without DLD, controlling for age, sex, and SES. In combination, the four cognitive factors underlying memory capacity accounted for 93% of the total variance in language in the TD group and 96% of the total variance in language in the DLD group. However, the magnitude of the relationship between WM and language was significantly larger for the TD group than the DLD group. Taken together, these findings are consistent with the broad definition of DLD as encompassing deficits in a wide range of cognitive and linguistic abilities. At the same time, these results are suggestive of a unique relationship between WM capacity and language ability in children with DLD that was not observed in the TD controls. Whether such a relationship is causal is not yet known. New experimental investigations are needed to test specific hypotheses about potential mediating or moderating roles of cognitive abilities, most specifically WM and LTM (conceptualized broadly as an unlimited associative representational system), in language development and language intervention in children with DLD.

## Data Availability Statement

The raw data supporting the conclusions of this article will be made available by the authors, without undue reservation.

## Ethics Statement

The studies involving human participants were reviewed and approved by Institutional Review Boards at Utah State University, Ohio University, San Diego State University, and the University of Texas at Dallas. Written informed consent to participate in this study was provided by the participants’ legal guardian/next of kin.

## Author Contributions

RG, JM, and JE designed the study and collected the data. SS analyzed the data. RG drafted the manuscript. All authors reviewed and edited it. All authors contributed to the article and approved the submitted version.

## Conflict of Interest

RG receives royalties from the sale of the Test of Narrative Language-2, which was administered to the children in this study. The remaining authors declare that the research was conducted in the absence of any commercial or financial relationships that could be construed as a potential conflict of interest.

## Publisher’s Note

All claims expressed in this article are solely those of the authors and do not necessarily represent those of their affiliated organizations, or those of the publisher, the editors and the reviewers. Any product that may be evaluated in this article, or claim that may be made by its manufacturer, is not guaranteed or endorsed by the publisher.
